# Predicting Trajectories of Hospitalizations in U.S. Older Adults with Newly Diagnosed Heart Failure

**DOI:** 10.1093/geroni/igaf122.3644

**Published:** 2025-12-31

**Authors:** Hanzhang Xu, Radha Dhingra, Bradley Hammill, Scott Lynch, Jessica West, Michael Green, Lesley Curtis, Matthew Dupre

**Affiliations:** Duke University, Durham, North Carolina, United States; Duke University School of Medicine, Durham, North Carolina, United States; Duke University School of Medicine, Durham, North Carolina, United States; Duke University, Durham, North Carolina, United States; Duke University School of Medicine, Durham, North Carolina, United States; Duke University School of Medicine, Durham, North Carolina, United States; Duke University School of Medicine, Durham, North Carolina, United States; Duke University School of Medicine, Durham, North Carolina, United States

## Abstract

Heart failure (HF) remains the leading cause of hospitalizations among older adults. Little is known about the longer-term risks of hospitalizations and whether high-risk patients can be identified at the time of HF diagnosis. We analyzed a national sample of Medicare beneficiaries with newly-diagnosed HF between 2010-2015. Patients were then followed for up to 5 years to identify all-cause hospitalizations after their initial diagnosis. Group-based trajectory models were used to identify patients with differing trajectories of hospitalizations and LASSO regression was used to identify patients’ characteristics at the time-of-diagnosis to predict their trajectory of hospitalizations. Model calibration was evaluated graphically (observed vs predicted) and discrimination was assessed using c-statistics. Of 84,597 beneficiaries (mean age: 77.4 [± 7.1] years and 58.3% male), we identified four distinct trajectories of hospitalizations over follow-up: Group 1 (n = 19,340; 22.9%) had “low risks” of hospitalization, Group 2 (n = 53,922; 57.9%) had elevated risks of admission shortly after diagnosis (“early risk” group), Group 3 (n = 5,035; 9.8%) had elevated risks at later stages of illness (“late risk” group), and Group 4 (n = 6,300; 9.4%) had consistently “high risks” of hospitalization. Models were well-calibrated predicting patients’ hospitalizations and discrimination (c-statistics) were 0.63 (Group 2), 0.67 (Group 3), and 0.77 (Group 4) when comparing to patients with low risks of hospitalization (Group 1). Predicting hospitalizations among beneficiaries improved in those who were diagnosed with HF at younger ages (≤70 years). Patients with high risks of hospitalizations could be identified at the time-of-diagnosis and should be targeted for interventions to improve HF management.

